# Real-World Toxicities and Outcomes of Pembrolizumab in Early-Stage Triple-Negative Breast Cancer

**DOI:** 10.3390/curroncol33090513

**Published:** 2026-08-27

**Authors:** Emmanuel Joran Boujeke, Aaron Catalan, Laurice Arayan, Alfred Patrick Mina, Christian Edward James-McDonald, Rasna Gupta, Swati Kulkarni, Abdullah Nasser, Deepro Chowdhury, Muriel Brackstone, John Mathews, Caroline Hamm

**Affiliations:** 1 Schulich School of Medicine and Dentistry, Western University, London, ON N6A 5C1, Canadacjamesmc@uwo.ca (C.E.J.-M.); caroline.hamm@wrh.on.ca (C.H.); 2 Windsor Regional Hospital, Windsor, ON N82 2X3, Canada; catalana@uwindsor.ca; 3 London Health Sciences Centre, London, ON N6A 5W9, Canada

**Keywords:** immunotherapy, pembrolizumab, breast cancer

## Abstract

**Purpose:** Pembrolizumab combined with chemotherapy is the standard of care for early-stage triple-negative breast cancer (TNBC) following KEYNOTE-522. However, real-world data on immune-related adverse events (irAEs) remain limited. We evaluated the incidence, severity, and clinical consequences of irAEs in a real-world cohort and compared outcomes with KEYNOTE-522. **Methods:** We conducted a retrospective cohort study of patients with stage II–III TNBC treated according to the KEYNOTE-522 regimen at two Ontario cancer centres between June 2022 and May 2024. Data on irAEs, treatment discontinuation, and pathological complete response (pCR) were collected and contextualized against trial outcomes. **Results:** Among 79 patients, the pCR rate was higher than that reported in KEYNOTE-522 (77.2% vs. 64.8%). irAEs were documented in 51.9% of patients, compared with 33.5% in the trial. Permanent discontinuation due to irAEs occurred in 22.8% of patients, compared with 15.7% reported in KEYNOTE-522. Most discontinuations occurred during the neoadjuvant phase. Conclusions: Real-world patients experienced higher observed rates of low-grade irAEs and treatment discontinuation than reported in KEYNOTE-522; however, differences in study design limit direct comparison. These findings highlight the need for optimized toxicity management and further study of the impact of treatment duration on long-term outcomes.

## Simple Summary

Pembrolizumab combined with chemotherapy has become a standard treatment for patients with early-stage triple-negative breast cancer. In this real-world study, higher rates of immune-related adverse events were observed than those reported in the KEYNOTE-522 trial, although severe toxicities remained uncommon. Many patients discontinued treatment because of toxicity, yet pathological complete response rates remained high. These findings highlight the importance of toxicity monitoring and the need for further research to better understand the impact of treatment discontinuation.

## 1. Introduction

Triple-negative breast cancer (TNBC) is a highly aggressive subtype of breast cancer characterized by the absence of estrogen receptor (ER), progesterone receptor (PR), and human epidermal growth factor receptor 2 (HER2) expression [1]. It accounts for approximately 10–15% of all breast cancers and disproportionately affects younger patients and those from racialized populations [2,3]. TNBC is associated with high rates of early recurrence, distant metastases, and poorer overall survival compared to other breast cancer subtypes [4].

Recent advances in immunotherapy have shown promise in improving outcomes for patients with early-stage TNBC. The KEYNOTE-522 trial was a pivotal phase III study evaluating the addition of pembrolizumab, a programmed cell death protein 1 (PD-1) inhibitor, to neoadjuvant chemotherapy [5]. The study demonstrated a significantly higher pathological complete response (pCR) rate in the pembrolizumab–chemotherapy group (64.8%) compared to the chemotherapy-alone group (51.2%). The regimen has also been shown to significantly improve event-free survival and overall survival [6,7]. These findings led to the adoption of pembrolizumab in combination with chemotherapy as the new standard of care for patients with stage II-III TNBC.

While clinical trials such as KEYNOTE-522 offer valuable efficacy and safety data, they are conducted with specific populations under controlled conditions that may not fully reflect real-world clinical practice [8]. In particular, real-world patients often present with more comorbidities, differing disease burden, and varied treatment adherence, which may influence the incidence and management of adverse events. Immunotherapy-related toxicities, including colitis, dermatitis, and endocrinopathies, are of particular concern, as they can be unpredictable, severe, and lead to early treatment discontinuation. Emerging real-world data suggest that the rates and severity of immunotherapy-related adverse events (irAEs) in clinical practice may exceed those expected based on controlled trials [9,10]. However, studies characterizing these toxicities in patients receiving pembrolizumab per the KEYNOTE-522 protocol remain limited. A better understanding of these toxicities and their impact on treatment in routine care settings is needed to optimize treatment and ensure patient safety.

In this study, we conducted a retrospective chart review of patients treated with chemoimmunotherapy for early-stage TNBC following the KEYNOTE-522 regimen. We aimed to characterize real-world toxicity profiles and treatment outcomes, with a focus on irAEs and treatment discontinuation rates. This study highlights the need for further research to better understand the impact of early immunotherapy discontinuation on treatment outcomes in TNBC.

## 2. Materials and Methods

### 2.1. Study Design and Setting

We conducted a retrospective observational cohort study at two regional cancer programs in Ontario, Canada: London Health Sciences Centre (LHSC), an academic centre, and Windsor Regional Hospital (WRH), a community centre. The study examined real-world treatment outcomes and irAEs among patients with early-stage TNBC treated according to the KEYNOTE-522 protocol.

### 2.2. Participants

Patients were eligible if they were diagnosed with clinical stage II–III TNBC and initiated on chemoimmunotherapy per the KEYNOTE-522 regimen between 1 June 2022 and 1 May 2024. Patients were required to have received at least one cycle of neoadjuvant pembrolizumab to be included in the analysis. This time frame was selected to coincide with the Ontario rollout of pembrolizumab for TNBC and allowed for a minimum follow-up of 6 months at the time of data collection to capture immunotherapy-related toxicities.

### 2.3. Data Collection

Study data were collected and managed using REDCap electronic data capture tools hosted at the University of Windsor. REDCap (Research Electronic Data Capture) is a secure, web-based software platform designed to support data capture for research studies, providing (1) an intuitive interface for validated data capture; (2) audit trails for tracking data manipulation and export procedures; (3) automated export procedures for seamless data downloads to common statistical packages; and (4) procedures for data integration and interoperability with external sources [11]. Collected variables included patient demographics and baseline clinical characteristics (age, date of diagnosis, tumour grade, nodal status, and disease stage according to the 8th edition of the American Joint Committee on Cancer [AJCC] staging system), treatment details (date of treatment initiation, pembrolizumab administration, and treatment discontinuation), and toxicity-related outcomes. IrAEs were categorized by type and graded according to the Common Terminology Criteria for Adverse Events (CTCAE) version 4.0. Adverse events were classified as irAEs based on documentation by the treating clinician indicating that the event was related to pembrolizumab. No separate adjudication of causality was conducted. IrAEs were evaluated by severity as any grade or high grade (grade ≥3) and were separated by treatment phase. Neoadjuvant toxicity rates were calculated using the full study cohort as the denominator. Adjuvant toxicity rates were calculated using only data from patients who received at least one dose of adjuvant pembrolizumab. Additional data included hospital admissions attributable to treatment toxicity (reason for admission, number of admissions, and length of stay), therapies administered for immunotoxicity, rates of treatment discontinuation due to toxicity, the proportion of planned immunotherapy received prior to discontinuation, and mortality attributable to immunotherapy-related adverse events.

### 2.4. Outcomes

The primary outcome was the incidence and severity of immunotherapy-related toxicities. Secondary outcomes included pathological complete response (pCR), treatment discontinuation due to irAEs, and hospitalization burden from toxicities. Pathological complete response was defined as the absence of residual invasive cancer in the breast and regional lymph nodes, with residual in situ disease permitted (ypT0/Tis ypN0). All 79 patients underwent surgery and had pathological response data available and were therefore included in the pCR analysis.

### 2.5. Data Analysis

Given the differences in study design, we summarized patient characteristics, treatment outcomes, and irAE data descriptively using frequencies and percentages. For KEYNOTE-522, baseline characteristics were obtained from the full pembrolizumab-arm population (N = 784), pCR results were obtained from the interim efficacy cohort (N = 401), and neoadjuvant and adjuvant irAE rates were obtained from the respective phase-specific safety populations (N = 781 and N = 547). For the real-world analysis, the full cohort (N = 79) was used for baseline characteristics, pCR outcomes, and neoadjuvant irAE rates, while adjuvant irAE rates were calculated among patients who received at least one dose of adjuvant pembrolizumab (N = 68). No formal statistical comparisons were performed between the real-world cohort and KEYNOTE-522.

### 2.6. Ethics

This study was approved by both the Windsor Regional Hospital and London Health Sciences Centre Research Ethics Boards (REB# 24-479 and 25-056, respectively).

## 3. Results

### 3.1. Participants

In total, 79 patients were included in the study. Baseline characteristics are presented in Table 1. Compared with KEYNOTE-522, the real-world cohort included a higher proportion of postmenopausal patients and patients with stage III disease. Other characteristics, including primary tumour classification and HER2 status, were similar between the cohorts.

### 3.2. Efficacy

Among the 79 patients, all of whom underwent surgery and had pathological response data available, 61 achieved pCR (ypT0/Tis ypN0), corresponding to a pCR rate of 77.2%. In comparison, the pivotal trial reported a pCR rate of 64.8%. The pCR rate was therefore numerically higher in our cohort compared to KEYNOTE-522.

### 3.3. Immunotherapy-Related Adverse Events

Immune-related adverse events (irAEs) were evaluated according to both overall (any-grade) and high-grade (grade ≥ 3) severity, as the latter represents clinically significant toxicities that often necessitate hospitalization, treatment interruption, or permanent discontinuation of therapy. The overall incidence of documented irAEs was 51.9% in our cohort, compared with 33.5% reported in KEYNOTE-522. Grade ≥ 3 events occurred in 16.5% and 12.9%, respectively. Below is a breakdown of specific types of irAEs by grade and treatment phase of occurrence.

### 3.4. Neoadjuvant Phase

During the neoadjuvant phase, irAEs occurred across multiple organ systems in both the real-world cohort and the KEYNOTE-522 cohort. Among any-grade events, the most common toxicities in the real-world population were dermatitis (15.2%), hypothyroidism (6.3%), and diarrhea or colitis (5.1%) (Table 2).

For grade ≥ 3 events (Table 3), the overall frequency of severe toxicities remained low in both cohorts. The most common grade ≥ 3 events in the real-world population were dermatitis (2.5%) and colitis (2.5%). One patient experienced grade 4 colitis, and no grade 5 toxicities were observed.

### 3.5. Adjuvant Phase

Of the 79 patients included in the study, 68 received at least one dose of adjuvant pembrolizumab and were included in the adjuvant toxicity analysis. During the adjuvant phase, the observed frequencies of any-grade irAEs were lower overall (Table 4). In the real-world study, the most common any-grade events were hypothyroidism (11.8%), diarrhea or colitis (7.4%), and dermatitis (7.4%). Neurotoxicity, which was not reported in KEYNOTE-522, occurred in 4.4% of patients.

Grade ≥ 3 toxicities during the adjuvant phase were uncommon in both cohorts (Table 5). The grade ≥ 3 events in the real-world study were neurotoxicity, hypothyroidism, colitis, adrenal insufficiency, and pneumonitis, each occurring in 1.5% of patients. No grade 4 or grade 5 toxicities were noted.

### 3.6. Treatment Discontinuation

Permanent discontinuation of pembrolizumab due to immune-related adverse events occurred in 18 patients (22.8%) in the real-world cohort, compared with 123 patients (15.7%) in the KEYNOTE-522 trial. In the real-world cohort, discontinuation occurred more often during the neoadjuvant phase, accounting for 61.1% of cases, with the remaining 38.9% occurring in the adjuvant setting. A similar pattern was observed in KEYNOTE-522, although discontinuations were more heavily concentrated in the neoadjuvant phase (85.4% vs. 14.6% in the adjuvant phase). The most common toxicities leading to discontinuation in the real-world population were colitis, dermatitis, and hypothyroidism. Specific causes of discontinuation were not reported in KEYNOTE-522. Among the 18 patients who permanently discontinued pembrolizumab because of an irAE, 15 (83.3%) achieved pCR, compared with 46 of 61 patients (75.4%) who did not discontinue. Table 6 summarizes the characteristics of patients who discontinued treatment in the real-world cohort.

### 3.7. Hospital Admission

Nine patients (11.4%) required hospital admission. Of these, 8 patients required a single admission, with 7 cases attributable to immunotoxicity and 1 to febrile neutropenia. One patient was admitted twice: initially for immunotoxicity and subsequently for febrile neutropenia. The mean time from initiation of immunotherapy to first admission was 149.2 days (median 139, range 16–312). No deaths were attributed to immunotoxicity.

## 4. Discussion

In this retrospective real-world analysis, we evaluated treatment outcomes and immunotherapy-related adverse events in patients with early-stage triple-negative breast cancer treated according to the KEYNOTE-522 regimen and compared these findings with those reported in the clinical trial.

The pCR rate in our real-world cohort was 77.2%, compared with 64.8% reported in KEYNOTE-522. This finding contrasts with other real-world studies, which have generally reported lower pCR rates relative to the pivotal trial [12,13,14]. Notably, this finding occurred despite a higher proportion of stage III and node-positive disease compared with KEYNOTE-522, which may in part reflect differences in staging methodology, as the real-world population was staged according to the AJCC 8th edition whereas KEYNOTE-522 used the AJCC 7th edition. The high pCR rate despite frequent early discontinuation of pembrolizumab is an intriguing observation that may suggest substantial treatment benefit occurs during the neoadjuvant phase [15,16,17]. However, pCR alone does not establish the effects of reduced treatment exposure on long-term outcomes, and trials explicitly evaluating optimal treatment dose and duration are required before therapy de-escalation can be considered.

Across all treatment phases, irAEs were documented in 51.9% of our cohort, compared with 33.5% reported in KEYNOTE-522. This finding is consistent with evidence from several other real-world studies reporting higher rates of irAEs [18,19]. When examining the distribution of toxicities, rates during the neoadjuvant phase were largely similar between cohorts. Dermatologic toxicity was the only irAE with a notably higher observed rate in our cohort. Dermatologic toxicities are often among the earliest manifestations of immune activation and may therefore be more readily identified in real-world settings [20,21]. Rates of grade ≥ 3 toxicities remained low in both cohorts, suggesting that toxicity was driven primarily by lower-grade events that, although often classified as mild to moderate, are clinically relevant as they may prompt treatment interruption, corticosteroid use, or increased healthcare utilization [22]. During the adjuvant phase, higher observed rates of hypothyroidism and gastrointestinal toxicity were documented in our cohort than reported in KEYNOTE-522. We also observed the occurrence of immune-related adverse events that were not reported in KEYNOTE-522, notably arthritis and neurotoxicity. The persistence of low rates of grade ≥ 3 toxicities across both cohorts suggests that severe, life-threatening irAEs remain uncommon, consistent with the established overall safety of pembrolizumab.

Permanent discontinuation of pembrolizumab due to irAEs occurred in 22.8% of patients (18/79) compared with 15.7% reported in KEYNOTE-522. Similarly high rates of discontinuation have been reported in other real-world studies [23]. Although one study found that early discontinuation was associated with lower pCR rates [24], the pCR rate in our cohort was numerically higher among patients who discontinued pembrolizumab than among those who did not (83.3% vs. 75.4%). However, the relationship between discontinuation and pCR cannot be determined from these findings, particularly because only 18 patients discontinued treatment and 7 did so during the adjuvant phase, after pCR had already been established. The clinical implications of discontinuation are still uncertain but remain relevant. This is of particular importance in the post-neoadjuvant setting where patients may receive capecitabine, olaparib, and/or adjuvant pembrolizumab, and treatment is increasingly being tailored based on response [25,26]. Decisions regarding continued therapy must therefore balance its potential benefit against the risk of ongoing immunotoxicity.

This study has several limitations. The relatively small sample size and inclusion of patients from only two regional cancer centres may limit the generalizability of the findings. Furthermore, KEYNOTE-522 used the AJCC 7th edition staging system, whereas patients in our cohort were staged using the 8th edition. Differences between these staging systems may confound comparisons of stage distribution and pCR rates and should be considered when interpreting our findings. The retrospective design also limits direct comparison with the prospective KEYNOTE-522 trial and introduces the potential for information and reporting bias, as irAEs were identified through routine clinical documentation rather than prospectively collected and graded. Consequently, lower-grade or transient irAEs may have been underreported, while severe toxicities may have been overrepresented because they were more likely to be documented. Attribution of adverse events in real-world practice can also be challenging. Some toxicities classified as immunotherapy-related may instead have been related to chemotherapy or multiple contributing factors, potentially resulting in misclassification and overestimation of irAE rates.

In summary, this real-world analysis demonstrated favorable pathological response rates and frequent lower-grade irAEs among patients receiving pembrolizumab-based therapy for early-stage TNBC. Although the observed proportion of irAEs was higher than that reported in KEYNOTE-522, differences in study design, patient selection, and toxicity ascertainment limit direct comparison between the cohorts. Severe irAEs were uncommon; however, lower-grade toxicities frequently contributed to treatment interruption and discontinuation. These findings underscore the need for proactive toxicity monitoring in routine practice and for further research examining how treatment discontinuation and duration of pembrolizumab exposure affect event-free and overall survival.

## Figures and Tables

**Table 1:** Characteristics of Patients at Baseline in the Real-World Study and the KEYNOTE-522 Study.

Characteristics	Real-World (N = 79)	KEYNOTE-522 (N = 784)
Age		
Median (range)—yr	53 (27–79)	49 (22–80)
<65 yr—no. (%)	62 (78.5)	701 (89.4)
Menopausal status—no. (%)		
Premenopausal	29 (36.7)	438 (55.9)
Postmenopausal	50 (63.3)	345 (44.0)
Primary tumor classification—no. (%)		
T1 to T2	59 (74.7)	580 (74.0)
T3 to T4	20 (25.3)	204 (26.0)
Nodal involvement—no. (%)		
Positive	46 (58.2)	405 (51.7)
Negative	33 (41.8)	379 (48.3)
Overall disease stage—no. (%)		
Stage II	41 (51.9)	590 (75.3)
Stage III	38 (48.1)	194 (24.7)
HER2 status score—no. (%)		
0–1	60 (75.9)	595 (75.9)
2+	19 (24.1)	188 (24.0)

**Table 2:** Immune-Related Adverse Events During the Neoadjuvant Phase (Any Grade).

Event	Real-World (N = 79)	KEYNOTE-522 (N = 781)
Arthritis	2 (2.5%)	0
Neurotoxicity	1 (1.3%)	0
Dermatitis	12 (15.2%)	34 (4.4%)
Hypothyroidism	5 (6.3%)	107 (13.7%)
Colitis	4 (5.1%)	13 (1.7%)
Myositis	2 (2.5%)	3 (0.4%)
Renal/Nephritis	1 (1.3%)	7 (0.9%)
Adrenal insufficiency	1 (1.3%)	18 (2.3%)
Hepatitis	1 (1.3%)	11 (1.4%)
Pneumonitis	1 (1.3%)	10 (1.3%)
Hyperthyroidism	0	36 (4.6%)
Diabetes	0	2 (0.3%)
Hypophysitis	0	14 (1.8%)

**Table 3:** Immune-Related Adverse Events During the Neoadjuvant Phase (≥Grade 3).

Event	Real-World (N = 79)	KEYNOTE-522 (N = 781)
Arthritis	1 (1.3%)	0
Neurotoxicity	0	0
Dermatitis	2 (2.5%)	30 (3.8%)
Hypothyroidism	0	3 (0.4%)
Colitis	2 (2.5%)	7 (0.9%)
Myositis	0	0
Renal/Nephritis	1 (1.3%)	7 (0.9%)
Adrenal insufficiency	0	10 (1.3%)
Hepatitis	0	9 (1.2%)
Pneumonitis	0	3 (0.4%)
Hyperthyroidism	0	2 (0.3%)
Diabetes	0	2 (0.3%)
Hypophysitis	0	8 (1.0%)

**Table 4:** Immune-Related Adverse Events During the Adjuvant Phase (Any Grade).

Event	Real-World (N = 68)	KEYNOTE-522 (N = 547)
Arthritis	1 (1.5%)	0
Neurotoxicity	3 (4.4%)	0
Dermatitis	5 (7.4%)	9 (1.6%)
Hypothyroidism	8 (11.8%)	10 (1.8%)
Colitis	5 (7.4%)	2 (0.4%)
Myositis	1 (1.5%)	1 (0.2%)
Renal/Nephritis	0	1 (0.2%)
Adrenal insufficiency	2 (2.9%)	3 (0.5%)
Hepatitis	0	0
Pneumonitis	1 (1.5%)	5 (0.9%)
Hyperthyroidism	0	4 (0.7%)
Diabetes	1 (1.5%)	1 (0.2%)
Hypophysitis	1 (1.5%)	0

**Table 5:** Immune-Related Adverse Events During the Adjuvant Phase (≥Grade 3).

Event	Real-World (N = 68)	KEYNOTE-522 (N = 547)
Arthritis	0	0
Neurotoxicity	1 (1.5%)	0
Dermatitis	0	4 (0.7%)
Hypothyroidism	1 (1.5%)	3 (0.4%)
Colitis	1 (1.5%)	1 (0.2%)
Myositis	0	0
Renal/Nephritis	0	0
Adrenal insufficiency	1 (1.5%)	0
Hepatitis	0	0
Pneumonitis	1 (1.5%)	2 (0.4%)
Hyperthyroidism	0	0
Diabetes	0	1 (0.2%)
Hypophysitis	0	0

**Table 6:** Characteristics of Patients Who Permanently Discontinued Treatment in the Real-World Study.

Characteristic	Value
Total number of patients with discontinuation	18 (22.8%)
Stage at diagnosis	
Stage II	8 (44.4%)
Stage III	10 (55.6%)
Mean % of planned immunotherapy received	46.2% ± 21.6
Timing of discontinuation	
Neoadjuvant phase	11 (61.1%)
Adjuvant phase	7 (38.9%)
irAEs leading to discontinuation	
Colitis	8 (44.4%)
Dermatitis	5 (27.8%)
Hypothyroidism	3 (16.7%)
Adrenal insufficiency	1 (5.6%)
Arthritis	1 (5.6%)
Pathological complete response (pCR)	15 (83.3%)

## Data Availability

The datasets generated during and/or analysed during the current study are not publicly available due to institutional privacy policies and the inclusion of potentially identifiable patient health information but are available from the corresponding author on reasonable request and with appropriate institutional approval.

## Abbreviations

The following abbreviations are used in this manuscript:

TNBC	Triple-Negative Breast Cancer
KEYNOTE-522	Name of the Phase III Pembrolizumab Clinical Trial in Early-Stage TNBC
irAE/irAEs	Immune-Related Adverse Event(s)
pCR	Pathological Complete Response
PD-1	Programmed Cell Death Protein 1
ER	Estrogen Receptor
PR	Progesterone Receptor
HER2	Human Epidermal Growth Factor Receptor 2
LHSC	London Health Sciences Centre
WRH	Windsor Regional Hospital
REDCap	Research Electronic Data Capture
AJCC	American Joint Committee on Cancer
CTCAE	Common Terminology Criteria for Adverse Events
REB	Research Ethics Board
